# Preoperative Left Ventricle End Diastolic Volume Index as a Predictor for Low Cardiac Output Syndrome After Surgical Closure of Secundum Atrial Septal Defect With Small-Sized Left Ventricle

**DOI:** 10.3389/fped.2021.705257

**Published:** 2021-12-24

**Authors:** Budi Rahmat, Nurima Ulya Dwita, Putu Wisnu Arya Wardana, Oktavia Lilyasari

**Affiliations:** ^1^Pediatric and Congenital Heart Surgery Unit, National Cardiovascular Center Harapan Kita, Jakarta, Indonesia; ^2^Department of Thoracic, Cardiac and Vascular Surgery, Faculty of Medicine, Cipto Mangunkusumo Hospital, University of Indonesia, Jakarta, Indonesia; ^3^Department of Cardiology and Vascular Medicine, Faculty of Medicine, University of Indonesia, National Cardiovascular Centre Harapan Kita, Jakarta, Indonesia

**Keywords:** atrial septal defect, left ventricle end diastolic volume, low cardiac output syndrome, ASD closure, magnetic resonance imaging (MRI)

## Abstract

**Introduction:** Low cardiac output syndrome is one of the postoperative complications that are associated with significant morbidity and mortality after surgical closure of atrial septal defect (ASD) with small-sized left ventricle (LV). This study investigated whether preoperative left ventricular end-diastolic volume index (LVEDVi) could accurately predict low cardiac output syndrome (LCOS) after surgical closure of ASD with small-sized LV.

**Method:** This retrospective cohort study involved adult ASD patients with small-sized LV from January 2018 to December 2019 in National Cardiovascular Center Harapan Kita. Preoperative MRI data to assess the left and right ventricle volume were collected. A bivariate analysis using independent Student's *t*-test was done. Diagnostic test using receiver operating characteristic (ROC) curve was also done to obtain the area under the curve (AUC) value. The best cutoff point was determined by Youden's index.

**Result:** Fifty-seven subjects were involved in this study [age (mean ± SD) 32.56 ± 13.15 years; weight (mean ± SD) 48.82 ± 12.15 kg]. Subjects who had post-operative LCOS (*n* = 30) have significantly lower LVEDVi (45.0 ± 7.42 ml/m^2^ vs. 64.15 ± 13.37 ml/m^2^; *p* < 0.001), LVEDV (64.6 ± 16.0 ml vs. 85.9 ± 20.7 ml; *p* < 0.001), LVSV (38.97 ± 11.5 ml vs. 53.13 ± 7.5 ml; *p* < 0.001), and LVSVi (27.28 ± 8.55 ml/m^2^ vs. 37.42 ± 5.35 ml/m^2^; *p* < 0.001) compared to subjects who did not have post-operative LCOS (*n* = 27). ROC analysis showed that the best AUC was found on LVEDVi (AUC 95.3%; 95% confidence interval: 90.6–100%). The best cutoff value for LVEDVi to predict the occurrence of LCOS after surgical closure of ASD was 53.3 ml/m^2^ with a sensitivity of 86.7% and a specificity of 85.2%.

**Conclusion:** This study showed that preoperative LVEDVi could predict LCOS after surgical closure of ASD with small-sized LV with a well-defined cutoff. The best cutoff value of LVEDVi to predict the occurrence of LCOS after surgical ASD closure was 53.5 ml/m^2^.

## Introduction

Secundum Atrial Septal Defect (ASD) is one of the most common congenital heart defects in children and adults (1, 2). Krumsdorf et al. (3) showed that the incidence of ASD continues to increase each year (3). ASD represents 10% of congenital heart defect cases and 20–40% of congenital diseases in adults. According to the data from National Cardiovascular Center Harapan Kita (NCCHK) Indonesia, the prevalence of ASD in 2018 is 691 cases, of which 80 cases already had surgical closure, and the mortality rate was 7%.

ASD allows a left-to-right shunt leading to a volume overload in the right ventricle, which further causes pulmonary hypertension (4). As the volume load of the right ventricle increases, the resulting compression on the left ventricle by the increased pressure in the right ventricle leads to an underloaded left ventricle (5). Combination of these pathological processes will produce a small-sized left ventricle than the right ventricle. Insufficient filling volume from the left atrium to the small-sized left ventricle will impair the left ventricular diastolic function.

After the closure of ASD, the left-to-right shunt from the left atrium to the right atrium will stop and the blood will start to flow from the left atrium to the left ventricle. Due to volume underload prior to the ASD closure, a sudden increase of blood volume to the previously small-sized left ventricle after the surgical closure will cause a diastolic dysfunction leading to a circulatory failure (3). Hence, the left ventricle will be unable to pump blood to the systemic circulation and the oxygen delivery will decrease. Therefore, after ASD closure, a decrease in cardiac output may be observed and cause postoperative low cardiac output syndrome (LCOS), which is associated with significant morbidity and mortality (3, 6).

It is important to assess the left ventricle volume to avoid circulatory failure caused by a sudden increase in left ventricle volume load after surgical closure (5). Several non-invasive imaging modalities can be used to assess the left ventricle volume, one of which is cardiac magnetic resonance imaging (MRI) (7). MRI is the gold standard examination for volume measurement in both ventricles and therefore can assess the left ventricle end-diastolic volume index (LVEDVi) (8, 9) that reflects left ventricular function and volume. This parameter could be a novel value to predict the occurrence of low cardiac output syndrome by determining the left ventricle volume's tolerance toward the sudden blood flow increase after ASD closure.

The objectives of this study were to evaluate whether LVEDVi could be used as a predictor of LCOS after surgical closure of ASD with small-sized LV and to determine the cutoff value of LVEDVi that could still tolerate sudden blood flow increase after surgical closure of ASD with small-sized LV.

## Methods

### Study Design and Variables

This study was a retrospective cohort study conducted at National Cardiovascular Center Harapan Kita (NCCHK) from January 2018 until December 2019. The study was approved by the ethical research committee (IRB). The inclusion criteria were patients diagnosed with ASD who underwent surgical ASD closure and the preoperative echocardiography result showed a small-sized LV (the size of the LV is <50% of the size of right ventricle). These criteria were related to our institutional policy where the preoprative MRI is only obtained in patients based on the small-sized LV criterion. Preoperative MRI data such as right ventricle ejection fraction (RVEF), right venricle stroke volume (RVSV), right ventricle stroke volume index (RVSVi), right ventricle end systolic volume (RVESV), right ventricle end systolic volume index (RVESVi), right ventricle end diastolic volume (RVEDV), right ventricle end diastolic volume index (RVEDVi), left ventricle ejection fraction (LVEF), left venricle stroke volume (LVSV), left ventricle stroke volume index (LVSVi), left ventricle end systolic volume (LVESV), left ventricle end systolic volume index (LVESVi), left ventricle end diastolic volume (LVEDV), and LVEDVi were measured. The valvular disease was evaluated by echocardiography. Sign of pulmonary arterial (PA) hypertension was evaluated by the presence of pruning sign from x-ray examination, low flow of pulmonary vein, bidirectional/right-to-left ASD shunt, and D-shaped left ventricle from echocardiography. Only patients with estimated PA pressure higher than 40 mmHg underwent cardiac catheterization prior to surgery based on institution protocol. The exclusion criteria used in this study were ASD patients with severe pulmonary hypertension (mean pulmonary arterial pressure >45 mmHg) with high pulmonary artery resistance index (>4 Woods unit) after being given the lung vasodilator test, those who had other organ malformations, or those who had a re-operation after ASD closure.

Surgical ASD closure was done either by pericardial patch or by direct closure. Tricuspid valve repair was done with ring annuloplasty/Kay procedure on the patient who develops moderate/severe tricuspid valve regurgitation. Mitral repair was done on a patient with severe mitral regurgitation. Aortic cross-clamp time and cardiopulmonary bypass machine time were measured. The postoperative LCOS was assessed by a combination of clinical parameters and laboratory findings, such as the presence of a minimum of two clinical findings (tachycardia, oliguria, or poor peripheral perfusion) and/or a central venous saturation <60% (oxygen extraction ratio higher than 40%), with or without metabolic acidosis or increased lactate levels, resulting in an escalation of therapy. This syndrome was evaluated and confirmed by our cardiac intensivist.

### Data Collection

All data were collected retrospectively in February 2020. Demographic and pre-operative MRI data of the patient were obtained through the medical record. Post-operative clinical patient data were collected through medical and nursing patient record review.

### Statistical Analysis

Data were collected and then analyzed using SPSS software Version 20.0. The data normality was assessed using Kolmogorov–Smirnov. Independent Student's *t*-test was used for bivariate analysis with normal data distribution; otherwise, Mann–Whitney test was used as a non-parametric test. The receiver operating characteristic (ROC) curve was used as a diagnostic test analysis to get the cutoff point between the LVEDVi value and the LCOS cases. Thus, we obtained the cutoff points and area under the curve (AUC). The Youden index was used to identify the best cutoff point (J = Sensitivity + Specificity – 1).

## Results

### Patient Characteristics

Fifty-seven subjects were included in this study. Fifty (83.9%) out of 57 subjects were females. The mean age of the subjects was 32.5 years, with the youngest being 13 years old and the oldest being 65 years old. Most subjects were NYHA II and NYHA III (45.6 and 49.1%, respectively). Almost all (94.7%) subjects had tricuspid valve regurgitation and some were accompanied with either mild (21%) or moderate (14.5%) mitral valve regurgitation (Table 1).

**Table 1:** Characteristic of subjects.

**Patient characteristics**	**LCOS**	**Without LCOS**	**Total**	***p*-value**
	**(*n* = 30)**	**(*n* = 27)**	**(*n* = 57)**	
**Preoperative data**				
Age (years)	32.43 ± 14.19	32.93 ± 11.99	32.56 ± 13.15	0.887^t^
Weight (kg)	49.49 ± 14.56	48.07 ± 8.96	48.82 ±12.15	0.715^t^
**Sex**				$0.138^{{\text{X}}^{2}}$
Male (%)	2 (28.6)	5 (71.4)	7 (12.3)	
Female (%)	28 (56)	22 (44)	50 (87.7)	
Preoperative arrhythmias (%)	2 (100)	0 (0)	2 (3.5)	-
**NYHA**				
I (%)	0 (0)	0 (0)	0	
II (%)	11 (42.3)	15 (57.7)	26 (45.6)	c
III (%)	18 (64.3)	10 (35.7)	28 (49.1)	$0.143^{{\text{X}}^{2}}$
IV (%)	1 (33.3)	2 (66.7)	3 (5.2)	0.633^fe^
Ejection fraction	71.52 ± 8.42	68.15 ± 10.68	69.92 ± 9.61	0.716^t^
**Tricuspid valve regurgitation**				
None	3 (100)	0 (0)	3 (5.2)	
Mild (%)	14 (43.8)	18 (56.2)	32 (56.1)	c
Moderate (%)	8 (61.5)	5 (38.5)	13 (22.8)	$0.234^{{\text{X}}^{2}}$
Severe (%)	5 (55.6)	4 (44.4)	9 (15.7)	0.707^fe^
Tricuspid valve gradient	51.29 ± 41.96	41.96 ± 14.39	46.79 ± 18.14	0.051
**Mitral valve regurgitation**				
None	21 (58.3)	15 (41.7)	36 (63.1)	
Mild (%)	4 (33.3)	8 (66.7)	12 (21.1)	c
Moderate (%)	5 (62.5)	3 (37.5)	8 14.1)	$0.133^{{\text{X}}^{2}}$
Severe (%)	0 (0)	1 (100)	1 (1.7)	0.88^fe^
**Intraoperative data**				
ASD size (mm)	28.73 ± 7.60	28.71 ± 6.9	28.72 ± 7.24	0.992^t^
Mitral valve repair (%)	4 (40)	6 (60)	9 (15)	$0.415^{{\text{X}}^{2}}$
Tricuspid valve repair (%)	15 (62.5)	9 (37.5)	24 ()	$0.168^{{\text{X}}^{2}}$
CPB duration (min)	75.19 ± 25.53	59.58 ± 20.39	66.56 ± 23.79	0.148^t^
Aortic cross-clamp time (min)	34.5 ± 17.13	30.42 ± 14.18	32.16 ± 15.60	0.919^t^
**Postoperative data**				
Length of stay in ICU (day)*	2 (1.5–4)	1 (1–1)	1 (1–2)	<0.001^mw^
Duration of ventilator usage (h)*	21 (12.5–38.5)	10.5 (6.75–14)	13 (8–26)	<0.001^mw^
**Use of inotropes**				
1 inotropic (%)	8 (33.3)	16 (66.7)	24 (42.1)	c
2 inotropic (%)	11 (55)	9 (45)	20 (35.1)	$0.121^{{\text{X}}^{2}}$
3 inotropic (%)	11 (84.6)	2 (15.4)	13 (22.8)	$0.002^{{\text{X}}^{2}}$
Post-operative arrhythmia	17 (73.9%)	6 (26.1%)	23 (100%)	$0.006^{{\text{X}}^{2}}$
Stroke after surgery (%)	0	0	0 (0%)	-
Hospital length of stay (days)	8.13 ± 2.31	6.43 ± 2.64	7.31 ± 2.60	0.11^t^

* *Data are presented using median (25th quartile−75th quartile)*.

*t, Independent t-test; mw, Mann–Whitney test; X^2^, Chi-square test; fe, Fisher's exact test; c, reference*.

Seventy-six percent of the subjects underwent surgical ASD closure using a pericardial patch, while only few used only polypropylene suture. Nine (14%) subjects with mild and moderate mitral valve regurgitation had surgical ASD closure complemented by mitral valve repair using ring annuloplasty (Table 1).

There was no significant difference between the mean duration of cardiopulmonary bypass (CPB) machine and the mean duration of aortic cross-clamp (AoX) in patients with and without postoperative LCOS. The subjects were transferred to the intensive care unit (ICU) right after the surgery had been completed, and ventilator support was used to improve their postoperative conditions. The median duration of ventilator usage and the median length of stay in the ICU were significantly higher in patients with postoperative LCOS (*p* < 0.001) (Table 1). Thirteen subjects (21%) had pulmonary hypertension postoperatively. Based on use of inotropes, 24 (42.1%) subjects used one inotrope, 20 (35.1%) subjects used two inotropes, and only 13 (22.8%) subjects used three inotropes.

### Comparison Between MRI Parameters as a Predictor of Low Cardiac Output Syndrome

Bivariate analysis was done for MRI parameter values and LCOS occurrence after the surgical closure of ASD. MRI parameter values were distributed normally. It showed that the LVSV, LVSVi, LVEDV, LVEDVi, LVESV, and LVESVi values were significantly lower in patients with LCOS (*p* < 0.05) (Table 2). However, no significant difference was found between the right ventricle ejection fraction and volume parameter across the two groups.

**Table 2:** Association between MRI parameter values and the occurrence of LCOS.

**MRI**	**LCOS**		***p*-value**
	**Yes (*n* = 30)**	**No (*n* = 27)**	
LVEF (%)	62.0 ± 8.6	60.7 ± 9.4	0.582^t^
LVSV (ml)	3.97 ± 11.5	53.13 ± 7.5	<0.001^t^
LVSVi (ml/m^2^)	27.28 ± 8.55	37.42 ± 5.35	<0.001^t^
LVEDV (ml)	64.6 ± 16.0	85.9 ± 20.7	<0.001^t^
LVEDVi (ml/m^2^)	45.0 ± 7.52	62.91 ± 11.9	<0.001^t^
LVESV (ml)	26.0 ± 7.2	33.2 ± 15.2	0.021^t^
LVESVi (ml/m^2^)*	17.5 (14.1–21.6)	22.6 (15.7–27.7)	0.019^mw^
RVEF (%)	48.6 ± 12.1	50.1 ± 11.1	0.616^t^
RVSV (ml)	128.8 (98.8–148.4)	139 (101.8–178.7)	0.299^mw^
RVSVi (ml/m^2^)	94.9 (64.9–104.2)	100.1 (66.4–120.1)	0.388^mw^
RVEDV (ml)	263.38 ± 79.6	265.1 ± 72.6	0.926^t^
RVEDVi (ml/m^2^)	192.9 ± 68.0	186.4 ± 61.9	0.695^t^
RVESV (ml)*	127.8 (105.8–168.3)	135.1(98.3–168.3)	0.644^mw^
RVESVi (ml/m^2^)*	92.8 (70.3–114)	91.9 (63.9–125.7)	0.555 ^mw^

* *Data are presented using median (25th quartile−75th quartile). t, Independent t-test; mw, Mann–Whitney test*.

**Figure 1:**
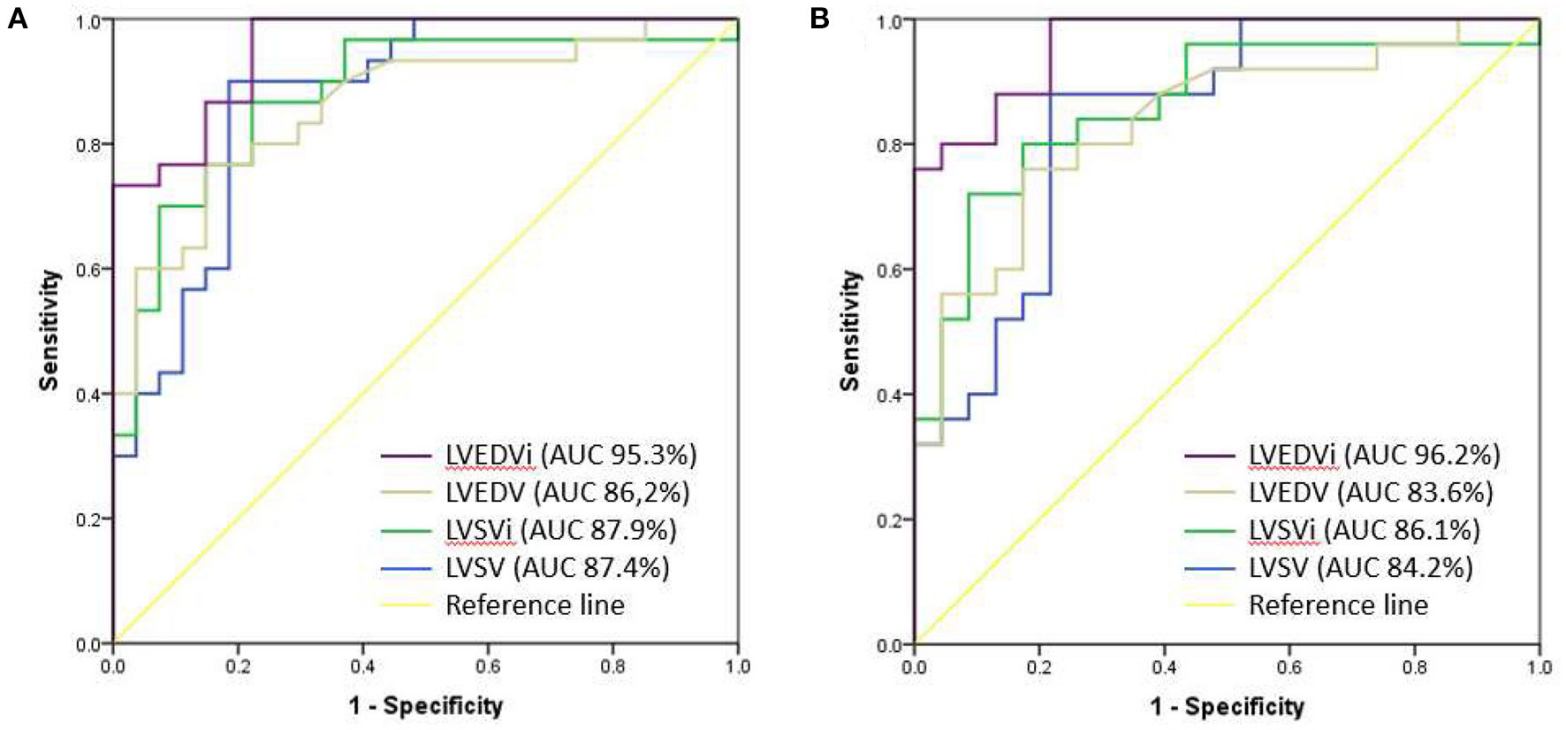
Comparison of AUC between LVEDVi (purple), LVEDV (brown), LVSVi (green) and LVSV (blue) for the occurrence of postoperative LCOS **(A)** and after exclusion of moderate-severe mitral valve regurgitation **(B)**.

**Figure 2:**
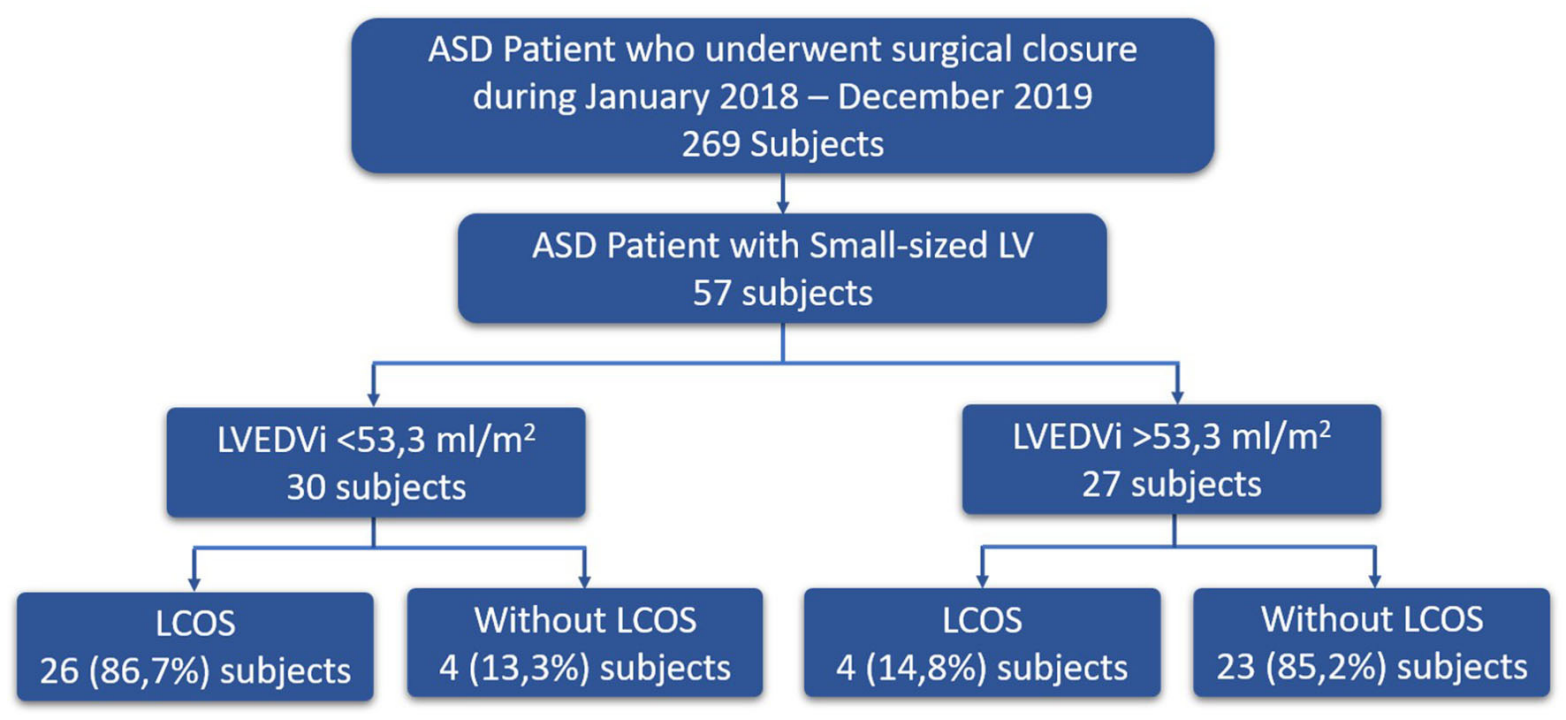
Consort diagram of ASD patient with small-sized left ventricle.

ROC curves were done between all cardiac MRI variables to predict the occurrence of LCOS. The acceptable AUC was found on LVEDVi (AUC 95.3%; 95% CI: 90.6–100%), LVEDV (AUC 95.3%; 95% CI: 76.7–95.8%), LVSVi (AUC 87.9%; 95% CI: 78.6–97.2%), and LVSV (AUC 87.4%; 95% CI: 78.2–96.6%) (Figure 1A). Meanwhile, we found no acceptable AUC between LVEF, LESV, LVESVi, RVEF, RVSV, RVSVi, RVEDV, RVEDVi, RVESV, and RVESVi with LCOS (Supplementary Figures 1–10). LVEDVi value had the strongest diagnostic power for LCOS occurrence compared to other MRI parameters. The optimal cutoff point values of each MRI parameter were obtained using the Youden index (Table 3). Based on these cutoffs, the best diagnostic value was found on LVEDV (cutoff point 53.3 ml/m^2^; sensitivity of 86.7%, specificity of 85.2%) followed by LVSV (cutoff point 48.7 ml/m^2^; sensitivity of 90.0%, specificity of 81.5%) (Figure 2).

**Table 3:** Comparison of AUC, sensitivity, and specificity between MRI parameter values.

**MRI parameter**	**AUC (%)**	***p*-value**	**95% CI**	**Cutoff value**	**Sensitivity**	**Specificity**
LVEF (%)	48.3	0.823	32.9–63.7	70.00	93.3	22.2
LVSV (ml)	87.4	<0.001	78.2–96.6	48.70	90.0	81.5
LVSVi (ml/m^2^)	87.9	<0.001	78.6–97.2	33.17	86.7	77.8
LVEDV (ml)	86.2	<0.001	76.7–95.8	75.55	76.7	85.2
LVEDVi (ml/m^2^)	95.3	<0.001	90.6–100	53.30	86.7	85.2
LVESV (ml)	65.5	0.045	51.0–80.0	31.05	76.7	55.6
LVESVi (ml/m^2^)	62.7	0.100	47.6–77.8	22.55	90.0	44.4
RVEF (%)	56.0	0.434	40.8–71.3	50.05	50.00	77.8
RVSV (ml)	58.0	0.299	42.7–73.4	132.85	63.3	63.0
RVSVi (ml/m^2^)	56.7	0.388	41.4–71.9	98.09	70.0	55.6
RVEDV (ml)	54.1	0.598	38.6–69.5	308.28	80.0	40.7
RVEDVi (ml/m^2^)	48.6	0.860	33.0–64.3	210.56	73.3	51.9
RVESV (ml)	49.3	0.924	34.0–64.5	157.92	73.3	37.0
RVESVi (ml/m^2^)	46.5	0.649	31.2–61.7	30.25	10.0	100

ROC analyses of LVEDVi, LVEDV, LVSV, and LVSVi value with the exclusion of moderate to severe mitral valve regurgitation subjects were done (Figure 1B) and optimal cutoffs of these variables were obtained (Supplementary Table 1). The best AUC was found on LVEDVi (AUC 96.2% CI 91.7–100%). We found that the best cutoff for LVEDVi was on the same value 53.3 ml/m^2^ with higher sensitivity (88.0%) and specificity (87.0%). However, the optimal cutoff point of LVSV showed lower with lower sensitivity (88.0%) and specificity (78.3%), given the same optimal cutoff (48.7 ml/m^2^).

## Discussion

LCOS occurs when there is a decrease in cardiac output caused by temporary myocardium dysfunction. LCOS may occur several hours after using a cardiac bypass machine, causing a decreased myocardium function due to increased cardiac output demand (10). If LCOS is not promptly recognized and treated, progression toward organ dysfunction is inevitable, leading to higher morbidity, prolonged intensive care and hospitalization, and mortality (11).

Out of 57 ASD patients in this study, more than half of the population (52.6%) had LCOS. However, Chandler et al. (12) reported that 25% of child population had a reduced cardiac index 6–8 h after an open heart surgery (12). The gap between the studies may have resulted from the particular inclusion ASD with small-sized LV, which is notably attributed to the late-presenting patients; therefore, higher proportion is expected. The mean value of age was 32.56 ± 13.15 years old, and our entire population showed signs of heart failure preoperatively (NYHA II 45.6%; NYHA III 49.1%; NYHA IV 5.2%). The average age of patients with NYHA II was older than that of patients with NYHA class III and IV. This indicates that the symptoms of heart failure in younger patients with large ASD were exacerbated by the lung overflow. The high occurrence of post-operative LCOS in older patients might be due to longer exposure of the underloaded left ventricle. Meanwhile, Chandler et al. did their study on infants without discussing the presence of heart failure before surgery.

Until now, no research had explained the significance of MRI parameters, especially LVEDVi, toward the postoperative LCOS incidence. Stephensen et al. reported that the MRI parameters in ASD patients for LVEDVi (82 ± 12 vs. 103 ± 14; *p* < 0.001) and LVESVi (35 ± 11 vs. 48 ± 8; *p* < 0.01) were significantly lower compared to the healthy subjects at rest (13). Compared to our study, in this ASD with small-sized LV population, we found that the LVEDVi value was lower especially in the LCOS group (45.0 ± 7.52). This could explain the high incidence of LCOS due to the underloaded LV preoperatively. Furthermore, moderate to severe mitral valve regurgitation combined with ASD could also affect the LV to become more small-sized due to an underpressured left ventricle with the backflow to the left atrium. However, in our subject, we had a similar proportion of mitral valve regurgitation in both LCOS and non-LCOS group. ROC analysis with the exclusion of moderate to severe mitral valve regurgitation subjects found that the best cutoff for LVEDVi was the same, 53.3 (AUC 96.2% CI 91.7–100%), with higher sensitivity (88.0%) and specificity (87.0%).

A small-sized LV that causes LCOS was explained by Schreiber et al. (14) in 14 patients who showed an abnormal intraventricular septum curvature at the end of the diastolic phase (14). Another theory explained that the left-to-right atrium shunt would cause a volume overload in both right atrium and ventricle, while the left ventricle will be underloaded (5). The left ventricle would be smaller or small-sized compared to the right ventricle, thus causing the left diastolic function. After ASD closure, the left-to-right shunt will cease and the blood flow will return to normal. The initially underloaded left flow would suddenly become normal; hence, the small-sized left ventricle would receive more blood. Left ventricular diastolic dysfunction will cause a circulatory failure (3). Consequently, the left ventricle will be unable to pump the blood to the systemic circulation, leading to LCOS. Therefore, it is necessary to assess the volume of the left ventricle before performing a surgical procedure.

To our knowledge, this study was never done before and may become a pilot study for further studies. Our study has shown a diagnostic test with an excellent result, which is applicable to clinical practices. Considering the cutoff value, physicians can determine the management strategy to anticipate the occurrence of LCOS post-surgical ASD closure. In our practice, the prediction of LCOS based on the preoperative measurement will give us the benefit of managing the availability of post-operative care resources. This will also give us guidance to do aggressive management of the small-sized left ventricle such as (1) leaving/creating a small ASD after complete closure of ASD intraoperatively (in order to reduce high left atrial pressure due to left ventricle dysfunction postoperatively), (2) utilizing left atrial pressure monitoring line during and after the operation to avoid left venticular failure, (3) placement of a temporary atrial pacemaker to increase the heart rate in order to avoid distention of the left venticle postoperatively, and/or (4) aggressive systemic afterload reduction with phosphodiesterase III inhibitors to improve LV function postoperatively.

The limitation of this study was that our subjects only focused on the ASD patient with small-sized LV, so the data implementation will be less representative of the overall population admitted for ASD closure. Regardless, this study provided a new insight related to management of patients with late-presenting ASD particularly with the small-sized LV condition. Some of our subjects had already achieved LCOS treatment on the small-sized LV through ACE inhibitor as the afterload reduction, which then becomes the confounding variable. Other confounding variables in our study were valvular regurgitations and post-surgical pulmonary hypertension. In this study, we only assessed one morbidity, which was LCOS. There are lots of morbidities that may occur after heart surgery such as arryhythmia, stroke, prolonged ventilator use, pulmonary hypertension, pulmonary edema, and death. Further studies with larger sample, multi-center and preferably the cohort are needed to evaluate and validate the LVEDVI cutoff value of this study.

## Conclusion

This study showed that LVEDVi could be used to predict the occurrence of LCOS after surgical closure of ASD with a well-defined cutoff (AUC 95.3%; 95% CI: 90.6–100%). The best LVEDVi cutoff value to predict LCOS after surgical closure of ASD was ≤53.3 ml/m^2^, with a sensitivity of 86.7% and a specificity of 85.2%. Validation of the achieved LVEDVi cutoff value is needed to assess LCOS after surgical closure of ASD at larger samples, and it is necessary to determine the LVEDVi cutoff value against other morbidities after surgical closure of ASD such as arryhthmia, stroke, duration of ventilator use, sepsis, pulmonary hypertension, pulmonary edema, and death.

## Data Availability Statement

The raw data supporting the conclusions of this article will be made available by the authors, without undue reservation.

## Ethics Statement

Written informed consent was obtained from the individual(s), and minor(s)' legal guardian/next of kin, for the publication of any potentially identifiable images or data included in this article.

## Author Contributions

BR, ND, and OL contributed to conception and design of the study. ND and PA organized the database. PA performed the statistical analysis. BR and PA wrote the first draft of the manuscript. BR, ND, PA, and OL wrote sections of the manuscript. All authors contributed to manuscript revision, read, and approved the submitted version.

## Conflict of Interest

The authors declare that the research was conducted in the absence of any commercial or financial relationships that could be construed as a potential conflict of interest.

## Publisher's Note

All claims expressed in this article are solely those of the authors and do not necessarily represent those of their affiliated organizations, or those of the publisher, the editors and the reviewers. Any product that may be evaluated in this article, or claim that may be made by its manufacturer, is not guaranteed or endorsed by the publisher.
