# Supplementary material for: Preoperative Left Ventricle End Diastolic Volume Index as a Predictor for Low Cardiac Output Syndrome After Surgical Closure of Secundum Atrial Septal Defect With Small-Sized Left Ventricle
Source: Front Pediatr. 2021 Dec 24;9:705257. doi: 10.3389/fped.2021.705257 (PMC8740283; doi:10.3389/fped.2021.705257)
Supplement: Supplementary file 1 [file Data_Sheet_1.docx]

**Supplementary Material**

**Table 1.** Comparison of AUC, sensitivity and specificity before and after exclusion of moderate-severe mitral regurgitation

| MRI parameter | AUC (%) | p-value | 95% CI | Cut-off value | Sensitivity | Specificity |
| --- | --- | --- | --- | --- | --- | --- |
| Before exclusion |  |  |  |  |  |  |
| LVSV (ml) | 87.4 | <0,001 | 78.2 – 96.6 | 48.70 | 90.0 | 81.5 |
| LVSVi (ml/m^2^) | 87.9 | <0,001 | 78.6 – 97.2 | 33.17 | 86.7 | 77.8 |
| LVEDV (ml) | 86.2 | <0,001 | 76.7 – 95,8 | 75.55 | 76.7 | 85.2 |
| LVEDVi (ml/m^2^) | 95.3 | <0,001 | 90.6 – 100 | 53.30 | 86.7 | 85.2 |
| After exclusion |  |  |  |  |  |  |
| LVSV (ml) | 84.2 | <0,001 | 72.9 – 95.4 | 48.70 | 88.0 | 78.3 |
| LVSVi (ml/m^2^) | 86.1 | <0,001 | 75.2 – 96.9 | 30.168 | 72.0 | 91.3 |
| LVEDV (ml) | 83.6 | <0,001 | 72.1 – 95.0 | 75.55 | 76.0 |  |
| LVEDVi (ml/m^2^) | 96.2 | <0,001 | 91.7 – 100 | 53,30 | 88.0 | 87.0 |


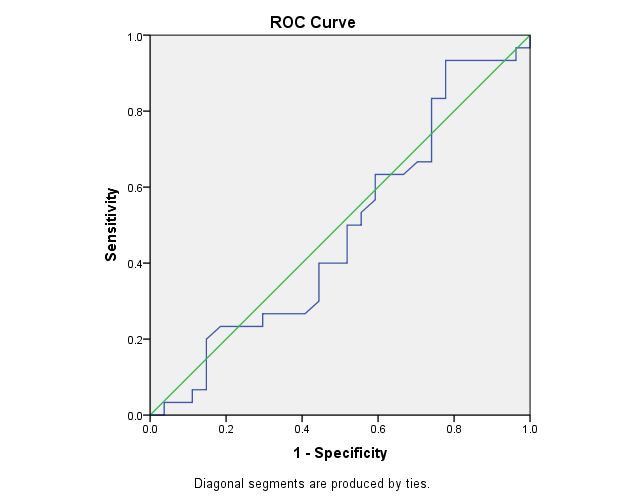


AUC 48.3% (CI 32.9 – 63.7%)

Best cut-off determined using Youden index was 70.0% (sensitivity of 93.3%, specificity of 22.2%)

**Figure 1.** ROC curve between LVEF and postoperative LCOS


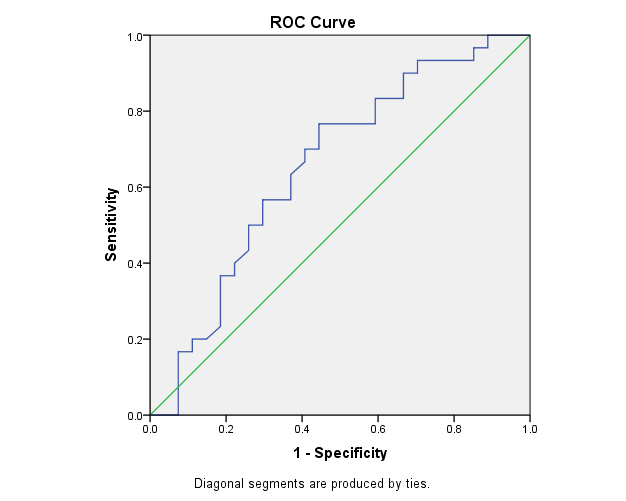


AUC 65.5% (CI 51.0-80.0%)

Best cut-off determined using Youden index was 31.05 ml (sensitivity of 76.7%, specificity of 55.6%)

**Figure 2.** ROC curve between LVESV and postoperative LCOS


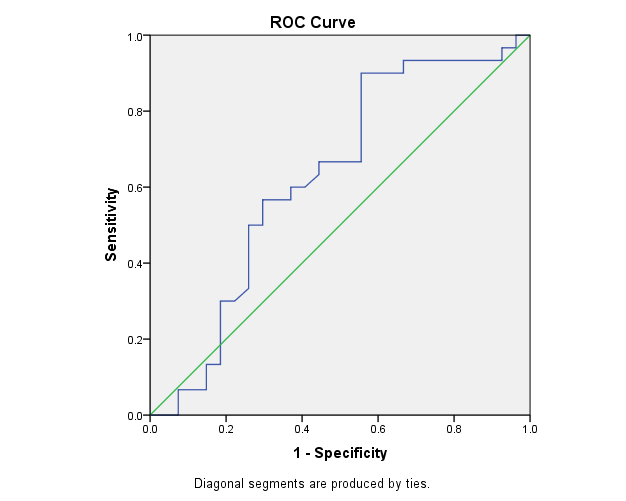


AUC 62.7% (CI 47.6-77.8%)

Best cut-off determined using Youden index was 22.55 ml/m2 (sensitivity of 90.0%, specificity of 44.4%)

**Figure 3.** ROC curve between LVESVi and postoperative LCOS


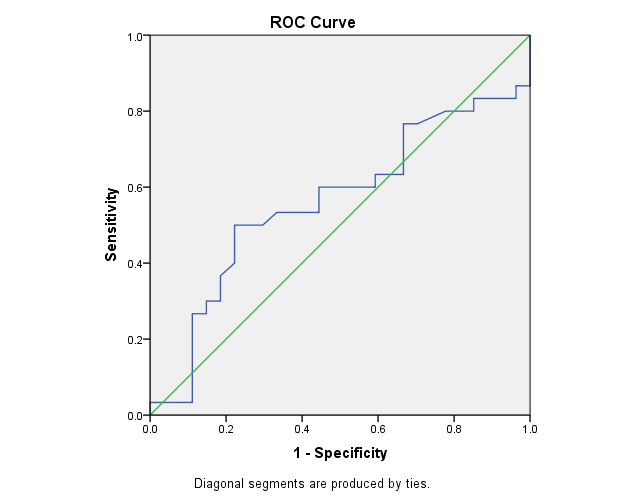


AUC 56.0% (CI 40.8 – 71.3%)

Best cut-off determined using Youden index was 50.05% (sensitivity 50.0% specificity 77.8%)

**Figure 4.** ROC curve between RVEF and postoperative LCOS


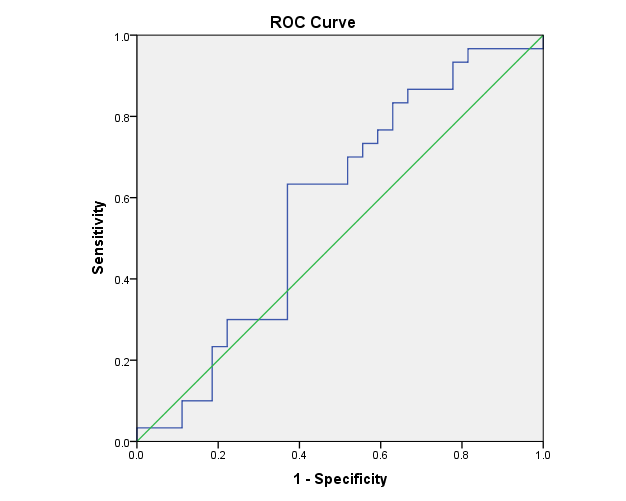


AUC 58.0% (CI 42.7 – 73.4%)

Best cut-off determined using Youden index was 132.85mL (sensitivity of 63.3%, specificity of 63.0%)

**Figure 5.** ROC curve between RVSV and postoperative LCOS


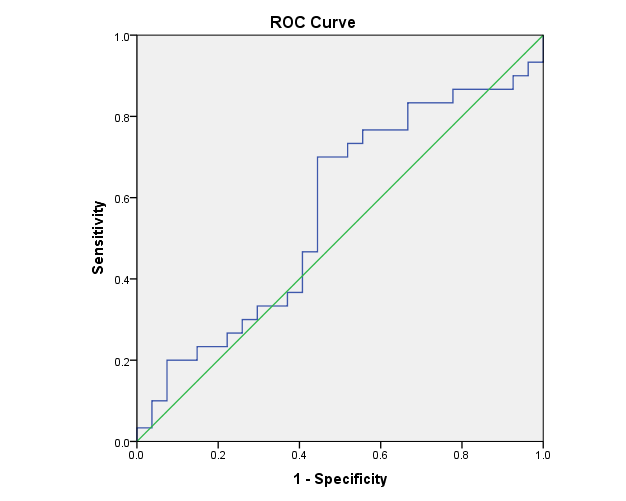


AUC 56.7% (CI 41.4 – 71.9%)

Best cut-off determined using Youden index was 98.09mL/m2 (sensitivity of 70.0%, specificity of 55.6%)

**Figure 6.** ROC curve between RVSVi and postoperative LCOS


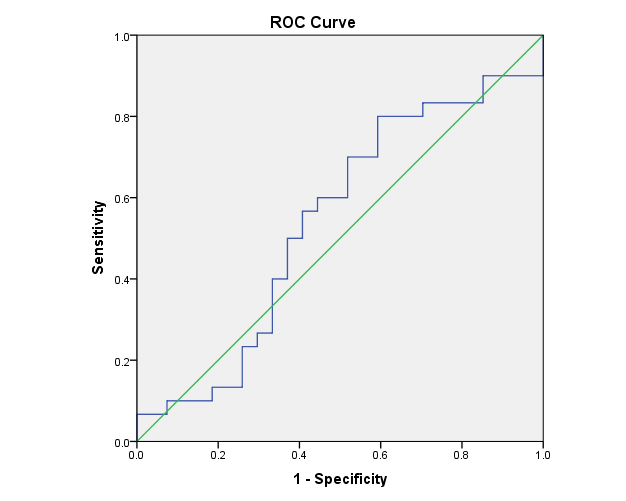


AUC 54.1% (CI 38.6 – 69.5%)

Best cut-off determined using Youden index was 308.82mL (sensitivity of 80.0%, specificity of 40.7%)

**Figure 7.** ROC curve between RVEDV and postoperative LCOS


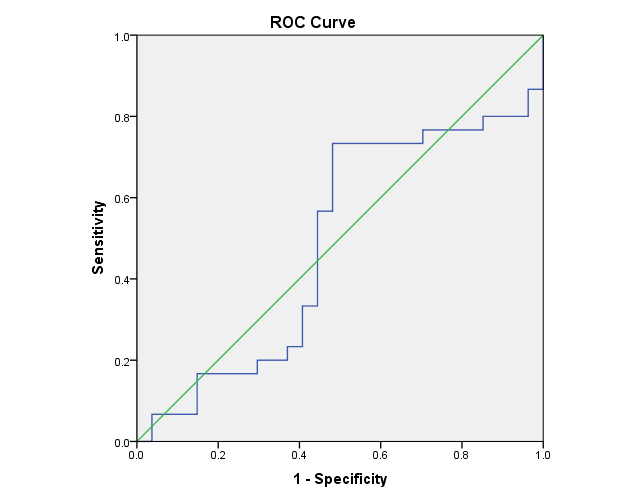


AUC 48.6% (CI 33.0 – 64.3%)

Best cut-off determined using Youden index was 210.56.mL/m2 (sensitivity of 73.3%, specificity of 37%)

**Figure 8.** ROC curve between RVEDVi and postoperative LCOS


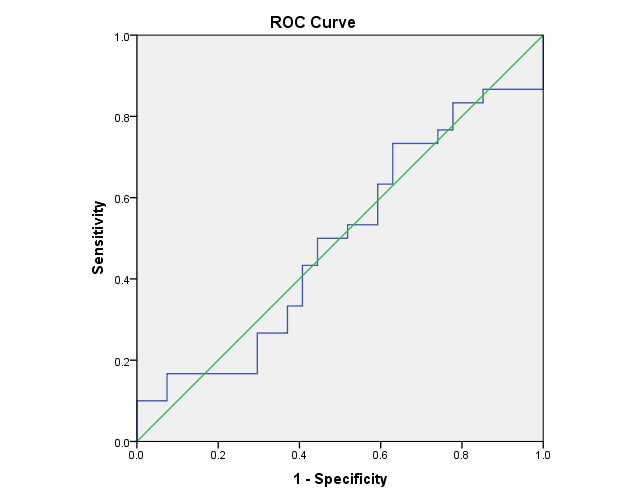


AUC 49.3% (CI 34.0-64.5%)

Best cut-off determined using Youden index was 157.92 (sensitivity 73.3% specificity 37.0%)

**Figure 9.** ROC curve between RVESV and postoperative LCOS


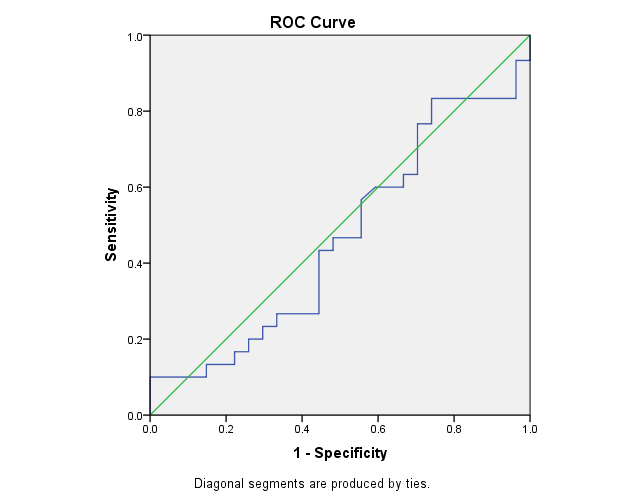


AUC 46.5% (CI 31.2-61.7%)

Best cut-off determined using Youden index was 30.25 ml/m2 (sensitivity 10% specificity 100%)

**Figure 10.** ROC curve between RVESVi and postoperative LCOS
